# Fernando de Castro and the discovery of the arterial chemoreceptors

**DOI:** 10.3389/fnana.2014.00025

**Published:** 2014-05-12

**Authors:** Constancio Gonzalez, Silvia V. Conde, Teresa Gallego-Martín, Elena Olea, Elvira Gonzalez-Obeso, Maria Ramirez, Sara Yubero, Maria T. Agapito, Angela Gomez-Niñno, Ana Obeso, Ricardo Rigual, Asunción Rocher

**Affiliations:** ^1^Departamento de Bioquímica y Biología Molecular y Fisiología, Instituto de Biología y Genética Molecular, Consejo Superior de Investigaciones Científicas, Universidad de ValladolidValladolid, España; ^2^CIBER de Enfermedades Respiratorias, Instituto de Salud Carlos III, Facultad de Medicina, Universidad de ValladolidValladolid, España

**Keywords:** Fernando de Castro, carotid body, arterial chemoreceptorss, sensory physiology, ion channels, transduction cascade

## Abstract

When de Castro entered the carotid body (CB) field, the organ was considered to be a small autonomic ganglion, a gland, a glomus or glomerulus, or a paraganglion. In his 1928 paper, de Castro concluded: *“In sum, the Glomus caroticum is innervated by centripetal fibers, whose trophic centers are located in the sensory ganglia of the glossopharyngeal, and not by centrifugal [efferent] or secretomotor fibers as is the case for glands; these are precisely the facts which lead to suppose that the Glomus caroticum is a sensory organ.”* A few pages down, de Castro wrote: “*The Glomus represents an organ with multiple receptors furnished with specialized receptor cells like those of other sensory organs [taste buds?]…As a plausible hypothesis we propose that the Glomus caroticum represents a sensory organ, at present the only one in its kind, dedicated to capture certain qualitative variations in the composition of blood, a function that, possibly by a reflex mechanism would have an effect on the functional activity of other organs… Therefore, the sensory fiber would not be directly stimulated by blood, but via the intermediation of the epithelial cells of the organ, which, as their structure suggests, possess a secretory function which would participate in the stimulation of the centripetal fibers.”* In our article we will recreate the experiments that allowed Fernando de Castro to reach this first conclusion. Also, we will scrutinize the natural endowments and the scientific knowledge that drove de Castro to make the triple hypotheses: the CB as chemoreceptor (variations in blood composition), as a secondary sensory receptor which functioning involves a chemical synapse, and as a center, origin of systemic reflexes. After a brief account of the systemic reflex effects resulting from the CB stimulation, we will complete our article with a general view of the cellular-molecular mechanisms currently thought to be involved in the functioning of this arterial chemoreceptor.

## INTRODUCTION: THE CAROTID BODY UNTIL FERNANDO DE CASTRO^1^

Hardowicus Wilhelmus Ludovicus Taube made his dissertation to obtain the greatest honors in Ars Medica the thirty first of January of 1743. The title of the dissertation was “De vera nervi intercostali origine” (The true origin of intercostal nerves), and in chapter 17 it reads “…, qui retro Carotides, ad ipsum interna and externa fecenditis angulum Ganglion minutum efficient, cujus ramuli, quantum video, in tunicis hujus arteriae desinunt,” what in a free translation would mean: “… behind the carotid arteries, where the internal and the external carotid arteries form their angle, Ganglion minutum forms, whose small twigs, as far as I can see end in the arterial walls.” Thus, ganglion minutum is the first name given to the carotid body (CB), which represented a small nervous structure located in the back of the carotid artery bifurcation. A few years later, Albrecht von Haller in his “Elementa physiologiae corporis humani” referred to the CB with the name of ganglion exiguum, as part of a plexus formed by branches of the superior cervical ganglion. Johann E. Neubauer, who provided the first drawing of the CB, referred to it as ganglion parvum; he depicted the CB as a small rectangular structure located in the angle of the carotid artery bifurcation but without any physical contact with the arteries themselves. Carolus S. Andersch, the discoverer of the petrosal ganglion which is the inferior or main ganglion of the glossopharyngeal nerve, named the CB, gangliolum intercaroticum, indicating in the name two important traits: its small size and its location. We want to explicitly state that even if Andersch discovered the inferior main sensory ganglion of the glossopharyngeal nerve (the superior, accessory or superior glossopharyngeal ganglion is named after his discoverer Johann Ehrenritter, the Ehrenritter ganglion), he did not describe any participation of this cranial nerve (the IX cranial pair) in the innervation of the CB.

In 1833–1834 August Franz Joseph Karl Mayer, the creator of the term histology, rediscovered the CB in man and several other species and named it ganglion intercaroticum. Besides describing it as a constant organ with a well defined relationship to the angle of the common carotid artery bifurcation through a small ligament, which in fact is a pad of fibroelastic tissue through which its arterial supply reaches the organ. In addition to its reddish color, its compactness and its size, like a grain of rice in man, the most important trait of the carotid he discovered was that, in addition of its connection with the superior cervical ganglion by one or more branches, the CB receives innervation from the glossopharyngeal through a fine branch that, running down parallel to the external carotid artery, ends up branching in the ganglion intercatoricum. We want to emphasize here to the fact that, in spite of the discovery of the innervation by the glossopharyngeal in the first third of the 19th century, it took almost a century to recognize the sensory nature of the innervation (de Castro, 1928).

Hubert von Luschka in 1862 found that the purported ganglion intercaroticum was not a ganglion, but a gland that he named glandula intercarotica. He provided many details about the size and anatomical variations in humans; he described a structure of the CB typical of a gland with glandular tubes and a close association with sympathetics, like the adrenal gland. Unfortunately, Luschka considered sympathetic innervation as the only nervous supply to the CB. He also described dispersed ganglion cells or small microganglia in his glandula intercarotica; Fernandode Castro described these ganglion cells in great detail in his 1926 article. It should be mentioned that Luschka was a very prestigious anatomist with multiple structures described and named after him (for a comprehensive list see Tubbs et al., 2011) and therefore his belief that the CB was a gland rooted deeply in the literature of that time. According to Noble et al. (1997) Luschka was the first to describe a CB tumor. A completely different point of view was proposed by Julius Arnold, son of Friedrich Arnold, the teacher of Luschka and his predecessor as anatomy professor in Tübingen. In his 1865 article, Julius Arnold presented the CB as formed by a complex net of small arteries, capillaries and venules that created a small round or oval shaped ball; it was not glandular tubes but rather convoluted vessels that gave this appearance. He named the CBs glomeruli arteriosi intercarotici, and the terminology has reached our days when we speak of glomic tissue or glomoid to refer to each of the nests of glomus cells (chemoreceptor cells) present in the CB along with the network of small vessels or capillaries surrounding each of them.

The glandular nature of the CB gained support from embryological studies, as Luschka himself and his disciples/followers proposed that it derived from the pharyngeal endoderm. Adams (1958) points out that Luschka’s proposal was based on an elementary mistake, as the CB was mistaken for a parathyroid gland. Arnold’s glomerular conception also received support from embryology as when in 1887, Kastchenko proposed that the CB was of mesodermal origin as it derives from a thickening of the adventitia in the vicinity of the origin of the internal carotid artery, close to the nodose ganglion. We do not want to proceed any further with this history before clarifying the embryological origin of the CB. At the beginning of 1970s, Le Douarin and coworkers developed a technique to create viable chimeras in avian embryos: grafts of the neural rhombencephalic primordium from 6 to 10-somite quail embryos were implanted in the homologous region of chick embryos of the same age, and after appropriate time the CBs of the host were histologically and immunohistochemically examined. Since quail cells are characterized by having interphase nuclear chromatin condensed in large masses while chicken cells have homogeneously distributed, uncondensed chromatin, it is possible to distinguish cells from each species; additionally, quail cells are dopamine-rich while chicken cells contain serotonin, making it possible to distinguish, on the basis of the spectrum of formaldehyde-induced fluorescence, the cells of quail origin which form part of the chicken CB. This type of studies allowed the French authors to demonstrate that, in fact, the parenchymatous cells, at least in the avian CB, derived from the neural crest, being neither endodermical nor mesodermical, but neuroectodermical (Le Douarin et al., 1972; Pearse et al., 1973).

It was the presence of biogenic amines in the CB cells that prompted Le Douarin to include them in APUD (amine content and/or amine precursor uptake and decarboxylation) series of endocrine polypeptide cells. The neuroectodermal origin of CB cells in mammals has also been demonstrated with different techniques, including genetic markers (e.g., Kameda, 2009). It is worth noting that neural crest cells migrate during embryogenesis according to their position along the embryo axis to give origin to many structures including most of the peripheral nervous system: the sympathetic and parasympathetic enteric ganglia, satellite glial cells in ganglia, dorsal root ganglia, Schwann cells, adrenal medullary chromaffin cells, and the CBs (see Hempleman and Warburton, 2013).

In retrospective, what we know today of the embryological origin of the CB (and adrenal medulla), provides a clear example of sound judgement, of the capacity of discernment of the scientists in the last part of the 19th and the early 20th century, when the technical support for their observations were rather limited. Thus, Kohn in 1900 recognized in his studies the observations made by Kastchenko on the thickening of the adventitia in the vicinity of the origin of the internal carotid artery, but added that this has nothing to do with the origin of the typical cells of the CB which arrive there along the sympathetic plexus growing in the carotid bifurcation. It would appear that Kohn asked himself how a structure should be named that is clearly neither a ganglion, nor a gland or a glomus. This answer was that since the CB, like adrenal medulla, is one of the organs connected to the sympathetic, it should be named paraganglion intercaroticum. This origin of today’s concept of paraganglion, as a group of cells, that not being neurons, are derived from the neural crest and are located in the vicinity of sympathetic or parasympathetic ganglia, was born in the past century. The first quarter of the 20th century was largely occupied by the discussion of the true nature of the CB as a paraganglion. The point was that the adrenal medulla, the best example of paraganglion, exhibited a very positive chromaffin reaction, i.e., a marked capacity to form yellow–brown precipitates when sections of the adrenal medulla were exposed to chromate and dichromate salts due to the adrenomedullary cells content in epinephrine and norepinephrine, but the CB showed, at best, a weak chromaffin reaction. Recognition of this fact, lead to the classification of paraganglia as chromaffin paraganglia (or sympathetic, as in the adrenal medulla) and non-chromaffin paraganglia (or parasympathetic, as in the CB). Thus, since according to Verna (1997) only those cells immunopositive to norepinephrine or its synthesizing enzyme, dopamine beta hydroxylase, exhibit chromaffin reaction, it follows that the classification of the CB as a chromaffin or non-chromaffin paraganglion could be species-related, according to the content of norepinephrine (see **Table 1**) and to the subjective appreciation of chromaffinity or pseudo-chromaffinity (is yellowship, chromaffin?). In establishing comparisons with the chromaffinity of the adrenal gland it should also be taken into account that the concentration of catecholamine in the adrenal gland is much higher than in the CB. It should be noted that some authors in the initial third of the 20th century also described the CB as a mixed paraganglia, or containing both chromaffin and non-chromaffin cells.

**Table 1:** Catecholamine content in the CB of several mammalian species.

Animal species	Dopamine (DA)	Norepinephrine (NE)	DA/NE
Cat	726 ± 114	697 ± 94	1.05
Rabbit	750 ± 78	143 ± 15	5.25
Rat	210 ± 24	45 ± 7	4.6
Mouse	225 ± 20	25 ± 3	9.0
Calf	598 ± 121	29 ± 4	20.6
Man	1.32 ± 0.4	1.63 ± 0.4	0.8^a^
	40 (at birth)–10 (young adult)^b^	–	–

*Data are expressed as nmole/g fresh tissue and have been obtained in our laboratory except for the data in humans. The data in humans are quite variable due to the fact that they have been obtained in necropsies with variable times elapsing from dead.*

a From Perrin et al. (1984).

b From Lack et al. (1985).

## THE 1926 ARTICLE OF FERNANDO DE CASTRO

**FIGURE 1:**
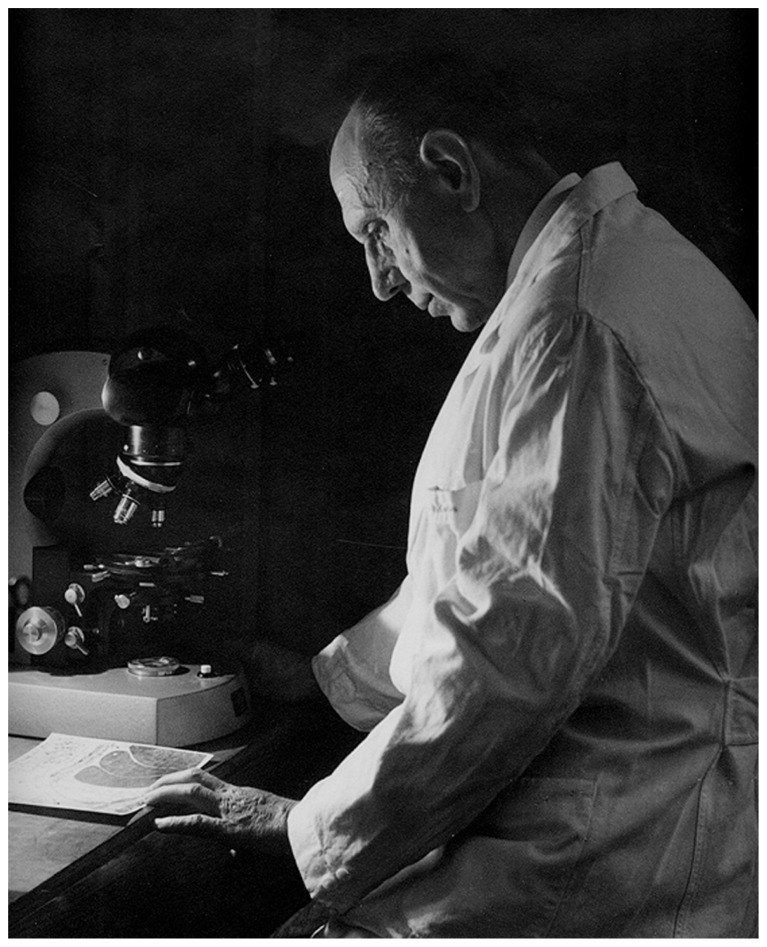
**Fernando de Castro (1896–1967)**. The picture was taken in 1967 (Courtesy of Fernando-Guillermo de Castro from the Archive Fernando de Castro).

In the Introduction to his 1926 article, de Castro (**Figure 1**) regretted the scarcity of studies devoted to the study of structure, innervation and function of the CB in contrast to the great profusion of embryological works. He pointed out that after Kohn the CB was considered by most researchers as a paraganglion, which like the organ of Zuckerkland or aortic paraganglion (located in the abdominal aorta in the vicinity of the origin of the inferior mesenteric artery or by the bifurcation of the aorta), the cardiac paraganglion or paraganglion of Wiesel (located in the fatty tissue around the left coronary artery among the branches of the cardiac plexus that supply the left auricle), and possibly also the coccygeal glomus gland of Luschka (located at the tip of coccyx) would represent supplementary adrenomedullary glands. de Castro also wrote that chromaffinity being the most conspicuous property of these paraganglia there was not unanimous agreement on the ability of the CB cells to reduce chromic salts. Nevertheless, there was general agreement on the spherical or ovoid form of the CB cells, their distribution contiguous to capillaries, and the richness of vessels and nerves of the organ. de Castro concluded the Introduction stating that little was known about the structure of the cells themselves, the nature and origin of the nerve fibers of the organ, and the way the nerve fibers end on the cells; these aspects were the targets of his 1926 study.

As to the methods of the paper (section I in the paper) we wish to underline a few details. (1) de Castro used in this study young and adult mice, rats, rabbits, cats, and dogs as well as mice and cat embryos at several developmental stages, mature human fetuses and human tissue from adult humans accidentally killed. (2) In the study of nerves de Castro used as staining procedure Cajal’s reduced silver nitrate method. In small animals and embryos, in order to prevent possible destruction or disturbances of the anatomical relationships, he fixed the entire head with several fixatives that included nitric acid as decalcifier, and serially sectioned the entire piece: this procedure allowed him to follow the nerves from their origin and to precisely define their anatomical relationships. (3) To study the cellular structure he used a new set of fixatives and staining procedures. (4) To experimentally study the innervation of the CB and the carotid region in general, he used cat and dog preparations in which the cervical sympathetic chain had been resected and/or the glossopharyngeal had been sectioned distally to the petrosal ganglion.

With this methodological armamentarium de Castro in 1926 made a series of salient findings that we shall try to faithfully summarize. From section II of the paper, devoted to the general organization of the innervation of the CB, we highlight the following aspects: de Castro wrote that in almost all studied species, the CB is surrounded by nerves which as a whole form a periglandular plexus (notice that de Castro follows the main trend in naming the CB as a gland). In mice the disposition of the fibers is much simpler and it is not feasible to talk of a truel periglandular plexus. Similarly, in humans, where the CB commonly is formed by several independent lobules (also common in calves; Sanz-Alfayate et al., 2001) dispersed in the conjunctive and fat tissue around the carotid sinus, there is not a true periglandular plexus. The fibers surrounding the CB, whether or not forming a true periglandular plexus, have three origins: (a) the greatest part comes from the superior cervical sympathetic ganglion and most of them are unmyelinated (i.e., postganglionic fibers) although there are some fibers of sympathetic origin which are middle sized myelinated (i.e., preganglionic that would terminate in sympathetic neurons located in the periphery of the CB or directly on chemoreceptor cells; see Verna, 1997); (b) the second contingent in quantitative importance comes from the glossopharyngeal via its intercarotid branch or *nerve intercarotidien* (the carotid sinus nerve, CSN), and is formed by myelinated fibers of middle size, although there are some myelinated fibers and some unmyelinated fibers; (c) finally, the smallest contingent is represented by filaments of fibers escaping from the pharyngeal branch of the vagus nerve. This organization of the periglandular plexus would explain two facts frequently disputed in recent literature: (1) for any given species, the levels of NE in the CB would vary from laboratory to laboratory according to their thoroughness on the dissection of the organ, i.e, depending on the elimination or not of the sympathetic fibers; (2) the effect of sympathectomy on the NE levels of the CB would vary from species to species according to their richness in NE-containing chemoreceptor cells (in the rat and rabbit, and probably in the mice, with few NE cells, sympathectomy would cause a great decrease in NE levels, while in the cat, the effect would be very modest; Gonzalez et al., 1997).

**FIGURE 2:**
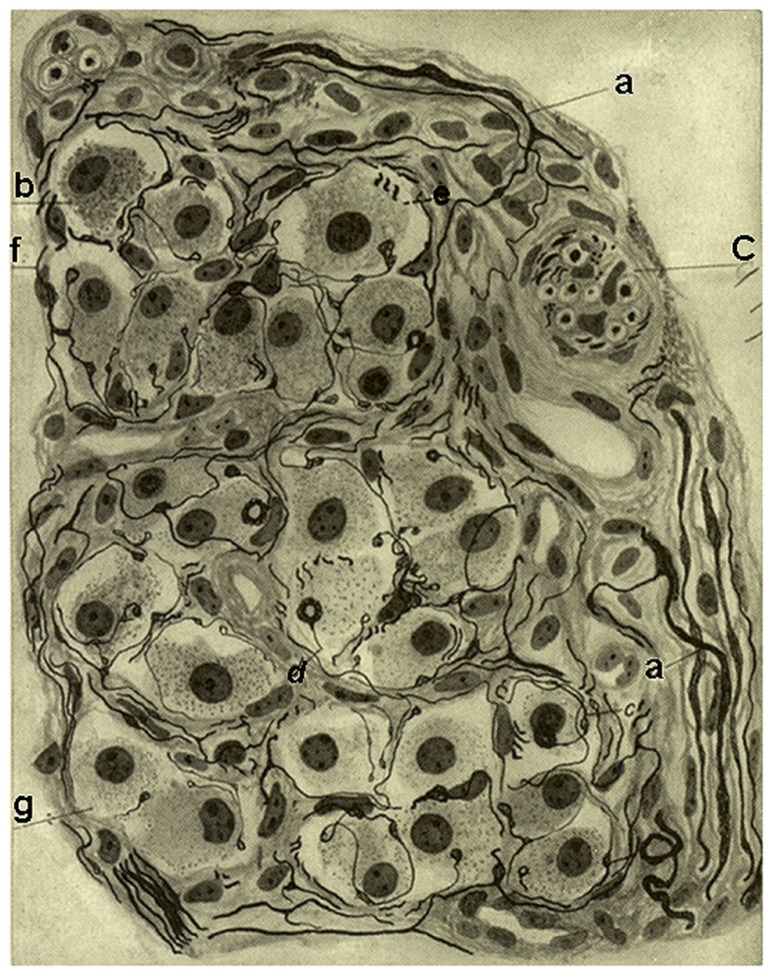
**Segment of a glomerulus of the intercarotid gland (the CB) of a young man**. c, nerve with myelinated and unmyelinated fibers; a, division of myelinated fibers; b, glandular(chemoreceptor) cell; e, glandular cell with a nerve ending in mallet; g, another cell with vacuolated cytoplasm; f, fiber with varicosities; d, nerve ending forming a thick ring. Stained by the Cajal’s reduced silver nitrate method (in de Castro, 1926; courtesy of Fernando-Guillermo de Castro from the Archive Fernando de Castro).

The interstitial plexus is formed almost exclusively by fibers of the CSN which penetrate the CB at different points, most commonly at superior or cephalic pole, and distribute and divide in the connective tissue that separates the clusters of parenchymatous cells; if the fibers are myelinated, it is commonly observed that, on dividing, they loose their myelin. The sympathetic fibers of the periglandular plexus do not penetrate the CB of the cat, dog, and man, but in mice, two or three thick sympathetic nerves penetrate and traverse the CB and, therefore, contribute to form the interstitial plexus. The fibers of the interstitial plexus, either isolated or in groups of up to eight or more, escape to form the periglomerular plexus which surrounds every cluster of parenchymatous cells. The periglomerular plexus forms on many occasions a true basket or nest around the cell clusters. Fibers of this plexus are both myelinated and unmyelinated and divide profusely and, upon dividing, myelinated fibers almost always loose the myelin sheath. From the periglomerular plexus, fibers commonly thin unmyelinated, penetrate cell clusters or glomeruli and frequently run associated with capillaries in the fine trabeculae of connective tissue forming the intraglomerular plexus (**Figure 2**). It is common that in this plexus fibers become very thin giving the impression that they end, but careful examination with adequate impregnation shows that they continue and widen to form polymorphic endings on the surface of chemoreceptor cells, from truly fine endings to small buttons or disks up to big calyx-shaped endings that almost completely envelope the entire cells (see the splendid images obtained in the serial section analysis with the electron microscope performed by Nishi and Stensaas, 1974). In the cat, rabbit, and dog the intraglomerular plexus is clearly discernable, being even more evident and complex in the human CB. In mice, where the intraglomerular connective tissue is absent and the parenchymatous cells contact each other, the intraglomerular plexus is less evident, fibers traveling freely among chemoreceptor cells.

On reading section II of de Castro’s paper, it strikes one that the author writes on no less than three or four occasions: the nerve endings do not penetrate the cell cytoplasm, but rather are always external to the cells and adapt to their surfaces. This sentence and its reiteration might sound meaningless today. Yet, it should be recalled that Cajal (de Castro’s mentor), the creator of the current theory of the nervous system organization known as the Neuron Doctrine (i.e., that the nervous system is made up of discrete individual cells, interrelated with one another by contact and not by continuity), had to defend his view against the proponents of the so-called reticularism (reticularist theory) which believed in the cytoplasmic continuity of nervous system cells, so that the cytoplasm of the neurons would form a continuous syncytium. The acrimonious attacks on the Neuron Doctrine are best exemplified by the Nobel Lecture given by Golgi in his sharing of the Nobel Prize with Cajal (see De Carlos and Borrell, 2007). Therefore, the statements of de Castro were meaningful in those years when the entity of neurons as independent cells was questioned.

Section III of the 1926 paper is devoted to the description of sympathetic (autonomic) neurons and microganglia in the CB and nervous plexuses just described. de Castro starts this section recognizing that Kohn has described these neurons/microganglia, and a few lines later states that the most important aspects of the biology of these neurons, i.e., the origin of their innervation and the destination of their axons, remain unknown. He asks himself, if these neurons innervate the parenchymatous cells of the gland (i.e., the chemoreceptor cells) or the CB blood vessels and other more distant structures? To answer these questions he used two main preparations: the perfused head of mice serially sectioned that allowed him to follow the trajectories of the fibers, and cat preparations which had been subjected to two different denervations 8–30 days before. The denervations consisted in either the removal of the cervical sympathetic chain or the section of the glossopharyngeal nerve cephalic to the origin of the CSN. His conclusions were: (1) Interspaced among the fibers of the periglandular and the interstial plexuses of the CB, at the origin of the CSN (particularly in the rat and mouse), and all along the length of the CSN there are autonomic neurons either isolated or in groups, forming true microganglia; as a whole these neurons are more frequent in the cat and dog than in the rat or mouse, and are very rare in man. (2) The preganglionic fibers of these neurons/microganglia have two origins, the brain stem (the medulla oblongata) and the spinal cord. Those fibers originating in the brain stem would belong to neurons located in the motor nuclei of the glossopharyngeal or vagus nerves, travel in the glossopharyngeal/CSN, and arborise on neurons located all along the CSN itself, in the periglandular plexus or inside the CB. The fibers originating from neurons located in the spinal cord come out via the superior cervical ganglion (forming part of the ganglio-glomerular nerves) and end up on neurons located in the periglandular plexus and in branches of the cervical sympathetics. The fibers with their origin in the brain stem degenerate on sectioning the glossopharyngeal and those originating in the spinal cord disappear after the removal of the sympathetic chain. (3) The axons from the autonomic neurons located in the CSN, on the surface, and inside the CB innervate the vessels of the CB and nearby structures; other contingents of fibers continue with sympathetic branches to innervate more distant structures.

We would not wish to close the description given by de Castro in 1926 of the autonomic neurons located in the glossopharyngeal and all along of the CSN without referring to the “efferent pathway.” The efferent pathway is a functional concept to describe the fact that stimulation of the peripheral cut end of the CSN inhibited chemoreceptor discharge recorded from single or few fiber filaments split off from the main nerve trunk (Almaraz et al., 1997). The point was that the anatomical substrate, the origin of the fibers supporting the efferent inhibition was unknown and persisted after every imaginable denervation procedure. Recent experiments have demonstrated that the axons from the autonomic microganglia constitute the efferent pathway. In a set of experiments Fidone’s laboratory re-described the autonomic neurons in the glossopharyngeal, in the origin of the CSN and along the CSN, and showed that these neurons are nitrergic which upon their activation release nitric oxide (and acetylcholine, implying that they are parasympathetic neurons). Nitric oxide and acetylcholine would cause vasodilation, with an additional supply of O_2_ to the CB and thereby causing the efferent inhibition; a direct effect of NO on chemoreceptor cells would also contribute to the efferent inhibition (see Wang et al., 1995). It remained, however, to be known how these neurons are physiologically activated. The obvious assumption was that signals activating them would come via the preganglionic fibers located in the brainstem, and it surely occurs that way, but quite recently Colin Nurses’s laboratory has made some brilliant experiments describing more direct forms of activation of these autonomic neurons. The Canadian authors confirmed the nitrergic-cholinergic nature of the autonomic neurons and showed that they are sensitive to hypoxia, so that hypoxia at the same time it augments the sensory activity provides a direct negative feed-back; they also showed that autonomic neurons express ATP receptors so that ATP released by chemoreceptor cells during hypoxic stimulation would activate the terminals of the autonomic neurons (or even the somas of neurons located inside the CB) promoting the genesis of nitric oxide which would be the ultimate effector. This last conclusion was obtained using a modification of the co-culture preparation developed in their laboratory. In this occasion they co-cultured clusters of chemoreceptor cells with autonomic neurons obtained from the glossopharyngeal-CSN and observed that application of ATP or hypoxia to the neurons caused a robust chemoreceptor cell hyperpolarization that was prevented by pre-incubation with NO scavengers and NOS inhibitors; additionally they showed that NO donors, but not ATP itself, was able to hyperpolarize chemoreceptor cells in clusters cultured without the autonomic neurons (see Campanucci et al., 2012).

**FIGURE 3:**
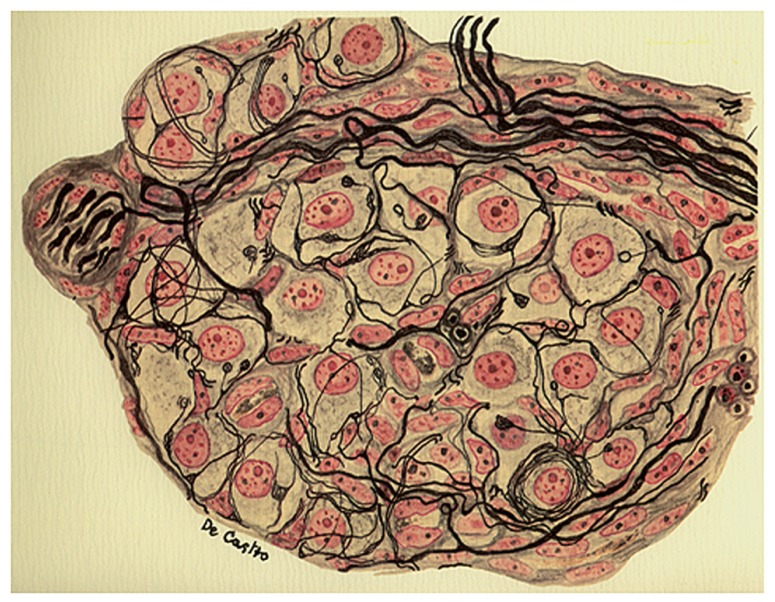
**Section from the intercarotid gland (the CB) of an adult cat 25 days after the surgical removal of the vertebral sympathetic chain**. The image evidences fascicles of myelinated nerve fibers and parenchymatous cells normally innervated. The preparation was stained by the Cajal’s reduced silver nitrate method and counterstained by the carmine method of Mayer (in de Castro, 1926, 1928, 1951; courtesy of Fernando-Guillermo de Castro from the Archive Fernando de Castro).

Section IV of the paper is entitled: “The intercarotid gland (the CB) is not innervated by the sympathetics of the paravertebral sympathetic chain: anatomical and experimental data.” Once again de Castro starts by referring to Kohn who proposed that the cells of the gland (the chemoreceptor cells) were innervated by the superior cervical sympathetic ganglion. In opposition to that, de Castro continues writing: in the cat there are many myelinated fibers (that could not be postganglionic sympathetic fibers), and what’s more, nobody has shown that all unmyelinated fibers, or only a part of them, come from the sympathetic system of the spinal cord or from the brainstem autonomic system. As in section III he used several experimental approaches: serial sectioning of the head of the mouse and preparations of cat and dog whose cervical sympathetic chain had been removed 15–30 days in advance. Referring to the mouse, de Castro writes: silver nitrate impregnation evidences that the nerve fascicles that originate in the superior cervical ganglion (the ganglioglomerular nerves) either go around the CB or traverse it, but none of the fibers arborise to penetrate in the cell clusters. Even further, it becomes evident that the fibers that penetrate the cell clusters or glomeruli arriving from the cephalic (brain stem) autonomic system are thicker and stain more intensely than the sympathetic fibers. As shown in **Figure 3**, representing a trustworthy copy of the microscope field of an operated cat, the removal of the cervical sympathetic chain of both sides does not alter the organization of intraglomerular plexus, i.e., the number of myelinated and unmyelinated fibers that penetrate the cell clusters and innervate the glandular (chemoreceptor) cells are undistinguishable from those seen in control cats. This implies that the innervation of chemoreceptor cells comes via the glossopharyngeal and the CSN from its origin in the autonomic system of the medulla oblongata. Readers must appreciate the erroneous bias in de Castro’s interpretation of his findings: since the CB purportedly was a gland, it must receive secretomotor motor innervation, and therefore the fibers which reach the glandular cells (chemoreceptor cells) via the CSN must have their origin in the brain stem (see below).

Section V of the paper is entitled: “Existence of a direct autonomic pathway for the intercarotid gland. Anatomical and experimental facts.” de Castro begins this section stating that he had already communicated to the Spanish Society of Biology that the intercarotid gland, the CB, appears to be innervated exclusively by the glossopharyngeal nerve via the CSN; the innervation is direct, without the interposition of an autonomic peripheral ganglion, a fact that is contrary to the admitted rule for the innervation of other glands and tissues controlled by the brain-stem, thoraco-lumbar, and sacral autonomic nervous system. In the paragraphs that follow he emphasizes the discrepancies that existed in the literature of that time, regarding the source of innervation of the CB and opposes to the view recently put forward by Drüner (1925). This last author, based on macroscopic studies in humans, inappropriate to solve the difficult problem of the CB innervation in the opinion of de Castro, proposed that innervation of the CB was sensory or centripetal and that CB itself represented the sensory organ responsible for the carotid baroreflex recently described by Hering.

The preparations used by de Castro to describe the innervation of the CB are those used in previous sections of the study, the head of mice and rat serially sectioned and cat and dog preparations whose cervical sympathetic chain had been removed or their glossopharyngeal had been sectioned 1–4 weeks in advance. After a meticulous description of the observations made in mice and rat he concludes that an autonomic pathway exists in the glossopharyngeal nerve that provides almost the entire wealth of fibers that constitute the CSN; these are efferent (centrifugal) secretory fibers devoted to innervating the intercarotid gland and are unique in the sense that they innervate the gland without the interposition of ganglion cells. de Castro estimated that these direct secretory fibers represented about 2/3 of total CSN fibers, the remaining 1/3 being preganglionic fibers that synapse with the autonomic neurons and microganglia described above, with an additional contingent of sensory fibers that innervate blood vessels that would be described in the next section. Our findings in the rat and mouse, continues de Castro, have been confirmed in the cat and dog as the extirpation of the cervical sympathetic chain does not appreciably alter the innervation of the gland while the section of the glossopharyngeal at its exit from the skull causes complete degeneration of the CSN, almost total disappearance of the interstitial and periglomerular plexus and complete loss of the intraglomerular plexus and its endings; occasionally after the section of the glossopharyngeal it is possible to see some myelinated axons which would be sensory fibers coming from the pharyngeal branch of the vagus nerve, from glossopharyngeal neurons displaced from their usual location (in the petrosal ganglion) or autonomic axons of the neurons located along the glossopharyngeal-CSN nerves, but in no case do they end on the glandular cells.

The description given by de Castro in this section is precise and it agrees with the descriptions given by modern morphologists (e.g., McDonald, 1981; Verna, 1997): nearly all the fibers ending on the glandular cells of the intercarotid gland (i.e., on the chemoreceptor cells of the CB) reach the organ via the CSN. Yet, de Castro was wrong in two aspects: in attributing the fibers an origin in the brain stem and in assigning them a secreto-motor function. Needless to say that de Castro corrected these two errors in his 1928 study, as we will see below. What lead de Castro to these erroneous conclusions? Obviously de Castro was short in his experimental observations. He performed sections on the glossopharyngeal at its exit from the skull, and this means distal to the glossopharyngeal sensory ganglion (petrosal ganglion), but he did not perform glossopharyngeal sections cephalic to the petrosal ganglion. In the cat (and also in the rat), the petrosal ganglion is located deep in the base of the skull, dorsomedial to the tympanic bulla really non-accessible and incapable of being seen from a ventral approach. That means that what indeed emerges from the base of the skull is the glossopharyngeal distal to the ganglion. As a consequence de Castro’s sections certainly caused the degeneration of fibers having their somas in the brain stem, but in addition all sensory neurons having their somas in the petrosal ganglion also degenerated. However, although de Castro was strictly faithful describing his observations, it is our opinion that he was biased in the interpretation by the current view that the CB was a gland, and obviously glandular cells must have an efferent secreto-motor innervation. In spite of the suggestion of Drüner, de Castro in 1926 had not yet considered that the CB could be a sensory organ, and certainly not the sensory element in the Hering’s reflex (see below), and therefore he did not search for a sensory innervation.

Section VI of the paper is devoted to the sensory innervation of the intraglandular vessels and to the innervation of the internal carotid artery. In the introduction to this section de Castro states that he is going to refer to the carotid sinus reflex described by Hering: mechanical stimulation by application of pressure of the region where the common carotid artery divides causes a diminution of the heart rate frequency and a vascular dilatation and, as a consequence, a fall in blood pressure. Already Hering had verified that the reflex remains intact after the removal of the superior cervical ganglion, but it disappears on sectioning the first branch of the glossopharyngeal (the CSN); additionally, Hering had also shown that the electrical stimulation of the central stump of the sectioned CSN mimicked exactly the mechanical stimulation of the carotid sinus region. Hering named this depressor nerve, “s*inusnerv,”* a name that de Castro considered inadequate (he preferred the name, nerve *intercarotiden*) because such a nerve would contain in addition to the sensory or centripetal fibers innervating the carotid sinus region, sensory fibers innervating the intraglomic vessels and the fibers innervating the CB itself which he thought at that time were autonomic centrifugal in nature.

Working in mice and rat de Castro describes that some (or most) of the sensory fibers mediating the depressor reflex (baroreceptor fibers), which arrive to the initial portion of the internal carotid artery via the CSN, come from the pharyngeal branch of the vagus nerve or even from the nodose ganglion itself. de Castro also described more complicated paths for the baroreceptor fibers of the vagus in their route toward the sinus region and assumes a comparable situation in the cat and dog. de Castro was surprised by the richness of innervation of this area, an aspect that nobody had noted before. The baroreceptor fibers mediating the depressor reflex are myelinated and upon reaching the origin of the internal carotid artery, which is a very densely innervated area, divide in the adventitia of the artery forming multi-shaped terminals in all species. In some cases, the fibers give rise to a few branches which form meniscus-shaped terminals of different sizes; on other occasions the fibers follow an arborisation pattern with progressively thinner branches which form small blebs in their trajectory or their end acquiring a varicose-like aspect. In all cases the terminals rest on the external elastic membrane of the artery. However, in a study using serial semithin and thin sections Böck and Gorgas (1976) confirmed the observations made by de Castro regarding the superficial disposition of the baroreceptor endings in mice, finding additionally that in the guinea pig the terminals enter the media and approach the innermost layers near the intima.

de Castro also performed denervation experiments in the cat and the dog. He found that removal of the superior cervical ganglion did not alter the innervation of the carotid sinus-origin of the internal carotid artery, and therefore gave histological support to the above mentioned physiological experiments performed by Hering. However, and contrary to the findings reported by Hering, de Castro found that sectioning of the glossopharyngeal early on its exit from the brain resulted in degeneration of the distal stump of the glossopharyngeal as well as degeneration of the CSN, but still the innervation of the internal carotid artery in its origin remained intact, implying that in large animals, as is the case in mice and rat, most of the depressor fibers would have their origin in the vagus nerve. This last conclusion was erroneous as de Castro himself recognized in later studies: most if not all baroreceptor fibers responsible for the carotid sinus baroreflex are in fact of glossopharyngeal origin having their soma in the petrosal ganglion (de Castro, 1940, 1951; see also Belmonte and Gallego, 1983).

**FIGURE 4:**
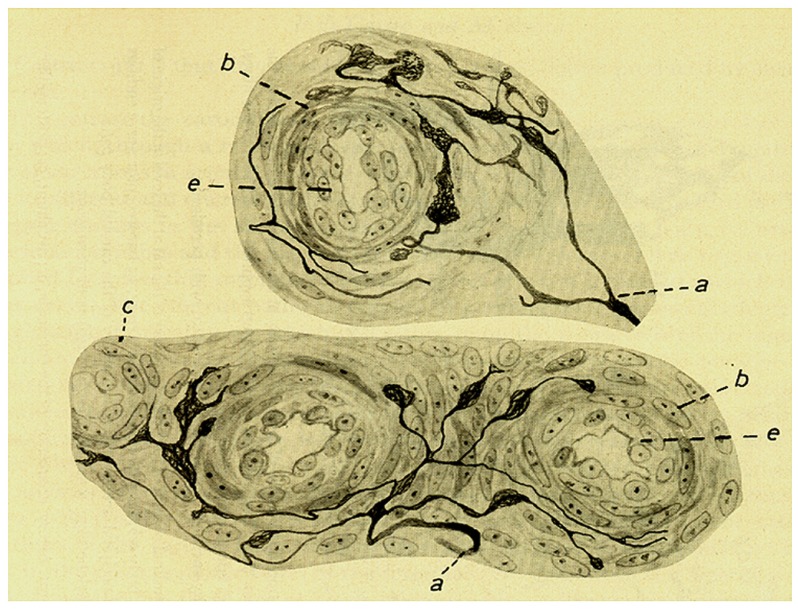
**Intra carotid body arterioles (e) receiving sensory innervation from two myelinated fibers (a) which arborise to terminate preferentially in the adventitia although some endings reach the muscular layer (b)**. (c) Smooth muscle cells. Branches form varicosities and terminate up forming small meniscus, buttons or hammer-like endings. Note that in the lower figure the fiber and its terminals are located in the angle formed by the branching of one artery. The preparation was stained by the Cajal’s reduced silver nitrate method (in de Castro, 1926; courtesy of Fernando-Guillermo de Castro from the Archive Fernando de Castro).

In this section de Castro also described a comparable innervation of the small intraglomic arteries and arterioles: myelinated fibers of glossopharyngeal origin penetrate the CB and from the interstitial plexus arrive to the innervated vessels. The fibers divide in the adventitia and form a nearly complete ring around the vessel to terminate forming varicose arborizations or meniscus, with the extension of the terminal arborisation being proportional to the diameter of the vessel; yet, the complexity of the terminals is smaller than that of their counterparts in the carotid sinus or the origin of the internal carotid artery. Although the sensory terminals most commonly are located in the adventitia most densely in branching points of the arteries, occasionally they reach the muscular layer or even approach the endothelium. de Castro underlines that these sensory fibers should not be confused with sympathetic fibers which terminate in the muscular layer. Regarding the function of this sensory innervation of the intraglomic vessels, de Castro considers that it would be different from the general baroreceptor (depressor) fibers present in the carotid sinus/origin of the internal carotid artery which support Hering’s reflex. He envisions the innervation of the intraglomic vessels as forming a reflex loop capable of regulating the functional activity of the CB (**Figure 4**). However, in his 1928 and 1951 studies de Castro concludes that the sensory system located in the intraglomic vessels also exerts an influence on the regularization of the arterial pressure, albeit smaller than that played by the carotid sinus innervation (see below under 1928 article).

Section VII of the article is devoted to the study of the chromaffin reaction of the CB. As we have mentioned above the chromaffinity of the CB was a much debated issue in the initial third of the 20 century, the discussion being linked to the concept of paraganglion, the embryological origin and the putative function of the CB itself. de Castro found that in the CB of man and cat fixed with formol-dichromate there are not cells exhibiting the typical brown chromaffin reaction (Henle’s reaction) as are seen in the suprarenal glands of the same individuals used as controls; at most in the CB cells there is a faint yellow and freely spread (non-granular) stain of the cytoplasm of the cells. In addition, the cells of the sections of the suprarenal glands also fixed with formol-dichromate when later stained with Giemsa stain acquire a granular aspect with a very striking greenish color, thought to be in agreement with other authors that grains would contain adrenaline, the secretory product of the cells. Contrary to that, the cytoplasm of CB cells after the same fixation and staining procedures acquires a pink tint. Since in addition CB cells stained in orange-red with Sudan III, de Castro concluded that the yellow staining of the cells did not represent a true, even if weak, chromaffin reaction, but rather a manifestation of the richness in lipoid material of these cells. Finally, CB cells did not exhibit the typical Vulpian reaction (development of a green color when a weak solution of ferric chloride is applied to the cells) seen in the cells of the suprarenal gland. At this point we want to refer to the study of Lever et al. (1959) which, along with additional observations, represents the full publication of their initial observation of the presence in chemoreceptor cells of osmiophile granular bodies (or dense-core granules or chromaffin vesicles) made in 1957 (Lever and Boyd, 1957). After using a significant number of histochemical tests they conclude that data strongly suggest the presence within the cytoplasm of chemoreceptor cells of a phenolic amine or some closely related chemical, because the relative order of staining intensities obtained with these tests was strikingly similar in the CB and in the adrenomedullary cells. The authors, also state that the intensity of staining was much less in the CB than in the adrenal gland, estimating in at least a factor of ten the difference in the intensity of the reactions between both tissues. Interestingly enough, the levels of catecholamine per unit weight in the adrenal gland are over one order of magnitude greater than in the CB. Agreeing with de Castro’s descriptions, Lever et al. (1959) also refer to the richness in lipoid like material and the presence of vacuoles in the cytoplasm of chemoreceptor cells.

de Castro also performed denervation and physiological experiments aiming to solve the issue of the chromaffinity of CB cells. He observed that denervation of adrenal glands in cats caused an augmentation of the chromaffinity in the denervated vs. the contralateral side with intact innervation. In agreement with other authors, he also observed that in normally innervated adrenal glands repeated injections of insulin (so as to produce a hypoglycaemic shock) caused a marked diminution, almost a depletion, of the brown grains (green after Giemsa staining) in the cytoplasm of adrenal gland cells. Since denervation of the glands prevented the action of insulin, de Castro concluded that the discharge of adrenalin was controlled by the central nervous system, and neither insulin nor the resulting hypoglycaemias have a direct effect on adrenomedullary cells. Contrary to the situation with the adrenal gland, the denervation of the CB did not cause any improvement in the chromaffin reaction, leading de Castro to conclude that there is no histochemical basis to consider the CB as a chromaffin organ capable of generating adrenaline. As pointed out by Adams is his monograph (Adams, 1958) it was precisely this study of de Castro that lead Kohn’s disciples to the coining of the concept of non-chromaffin or parasympathetic paraganglion. Ironically, when this concept began to be used in 1934, de Castro had already shown that the CB was a sensory organ rather than a supplementary gland. It should be noted, however, that the term parasympathetic paraganglia continues to be in use particularly by clinicians and pathologists to refer to tumoral masses of neural origin lacking an adrenergic trait, associated primarily to the vagus nerve, and usually located in the base of the skull, in the neck or in the mediastinum (Welander et al., 2011).

Section number VIII is devoted to the structure of the CB cells with a particular emphasis on mitochondria, Golgi apparatus (Golgi reticle) and lipoid material. The cells, which are disposed forming clusters, are frequently round or ovoid and on occasion they have processes that appear to anastomose. Part of the surface of the cells faces blood capillaries, this part of the cells being named the vascular or metabolic pole. The cell cytoplasm has a fine granular appearance and presents vacuoles either isolated or forming groups. They only have one nucleus although exceptionally they might have two. The nucleus is not centered, being usually displaced to the pole opposed to the capillaries, and the chromatin is usually diffuse although smaller cells have more condensed chromatin.

According to de Castro, in the cells of the CB nobody had studied before the chondriome or chondrioma, i.e., the mitochondrial content of the cells taken as a whole. We consider that the morphological description of mitochondria and additional organelles given by de Castro in those days has a limited value for today’s readers, as evidently ultrastructural analysis during the second half of the past century with the electron microscope has allowed the acquisition of very neat and precise images (see for example Verna, 1979). Nonetheless, there are certain observations which demonstrate the great capacity of discernment of de Castro. For example, during the description of mitochondria he states that circular mitochondria with a clear or empty center are not seen in the cells of the experimental animals, being occasionally noticeable in human cells, particularly if a long time has elapsed between death and the autopsy of the individuals. In other words, de Castro clearly noticed that late or poor fixation of tissues resulted in a loss of mitochondrial matrix. Another fact that strikes one on reading this section of the paper is the profusion of fixative and staining procedures used by de Castro to optimize the observations of particular organelles. Finally, also notable is the preoccupation transmitted by de Castro regarding the purported secretion of chemoreceptor cells. The notion behind this appeared to be: if indeed the CB is a gland, where is the secretion product formed? He stated not to have obtained experimental support to attribute either to mitochondria or to the Golgi apparatus the origin of the product of secretion. The pioneer work of Lever and Boyd (1957) demonstrated that chemoreceptor posses dense core granules and, as they suggested and we know today, they contain catecholamine. Dense core granules of chemoreceptor cells represent synaptic vesicles which secrete their content in response to the physiological stimulation of the CB not to the blood stream but rather to the synaptic spaces created by the cells and the sensory nerve endings innervating them (Iturriaga et al., 2009).

In the last section of the article de Castro makes some general conclusions that deserve to be mentioned. (1) Presently, we lack specific facts providing clues about the functional significance of the so-called intercarotid gland (the CB). (2) The CB is not a vital organ necessary for survival or for the correct development of the entire organism, as is the case for the thyroid gland. (3) On the other hand it cannot be admitted that the CB is a rudimentary or involutional organ as it persists for the entire life span of individuals without signs of degeneration or atrophy. (4) Rather, the complex innervation of the CB cells as well as the great vascularization of the organ and the profuse innervation of the CB vessels favors the notion of a very important role, perhaps assisting the depressor nerves of the carotid sinus in the regularization of arterial blood pressure through the secretion of a hypotensive substance. In the 1928 article, Fernando de Castro set the key bases that lead to satisfactory answers to these queries.

## THE 1928 ARTICLE OF FERNANDO DE CASTRO

There are two central themes in the planning or design of this study, which, by its own right, has originated a new area of physiology, namely the area of arterial chemoreception. In the first part of the study, de Castro develops the idea of the carotid sinus baroreceptor reflex as described by Hering and describes experiments on the structure and innervation of the carotid sinus area in several mammalian species giving full support to the notion that the origin of the reflex is the carotid sinus and its profuse innervation. The second part of the paper describes new denervations of the CB by cutting the rootlets of the glossopharyngeal at their exit from the brain stem, representing therefore a denervation of the CB cephalic to petrosal ganglion; this denervation procedure would leave intact the sensory innervation of the organ and cause degeneration of those fibers-endings having their somas in the brainstem. It is worth noting that in the title of 1926 study, de Castro refers to the intercarotid gland while in the 1928 he refers to the glomus caroticum.

As in the 1926 study, in this new work there is also a section on material and methods in which he states that to study the region of the common carotid artery bifurcation he has used material obtained from young men, monkeys, and calves as well as common laboratory animals from dogs to mice. He underlines the promptness in fixation of the tissues commonly in pyridine and the preferential use of Cajal’s reduced silver nitrate method to stain the fibers and their terminals. As an alternative fixative, de Castro provides the formula for his two-step fixation procedure in choral hydrate and somniphene solutions, and as alternative staining procedures he used the Bielschowsky’s silver and the Ehrlich-Dogiel methylene blue methods, emphasizing the need for careful handling of the last method to avoid the appearance of artifacts (formation of bubbles in the nerve fibers and terminals which may acquire the aspect of a string of beads).

Under the heading of macroscopic and microscopic anatomy of the carotid sinus, de Castro starts describing that in humans in the division of the common carotid artery, or more precisely in the origin of internal carotid artery, there is a constant and normal dilation or enlargement, the carotid sinus. Its external appearance is variable which has leaded some authors to classify them by their form. In laboratory animals, the sinus is very prominent. For example, in the cat whose internal carotid artery is very thin, the sinus has a large cone-shaped form with the base in the common carotid artery and with the internal carotid artery starting at the apex of the cone. As previously recognized by other authors, de Castro confirms the particular structure of the artery wall at the sinus level: it becomes thinner than the proximal and distal zones, this thinning being due to the absence of the media or muscular layer with an elastic layer and an adventitia well preserved (elastic segment). Since in the cow the internal carotid artery is absent (the common carotid artery gives origin to a thick eternal carotid artery, a glossofacial artery and the occipital artery), de Castro wanted to determine if in the area of the common carotid artery branching there was anything like the elastic segment of other mammals. He observed that indeed the occipital artery, which is responsible for the blood supply of the encephalon, presented dilation in its origin with a structure like that seen in the sinus-origin of the internal carotid artery of other mammals. In the guinea pig, which also lacks an internal carotid artery, it is also the occipital artery which is enlarged in its origin and presents the elastic segment (see Böck and Gorgas, 1976). However, in this same article the German authors observed that in the wall of the mouse carotid sinus a corresponding “elastic segment” is not evident. Similarly in the rat, according to Yates and Chen (1980), the vascular wall of the baroreceptor field in the internal carotid artery exhibits neither a marked dilation to form a carotid sinus nor histological differences in the intima and media compared to other parts of the carotid artery

**FIGURE 5:**
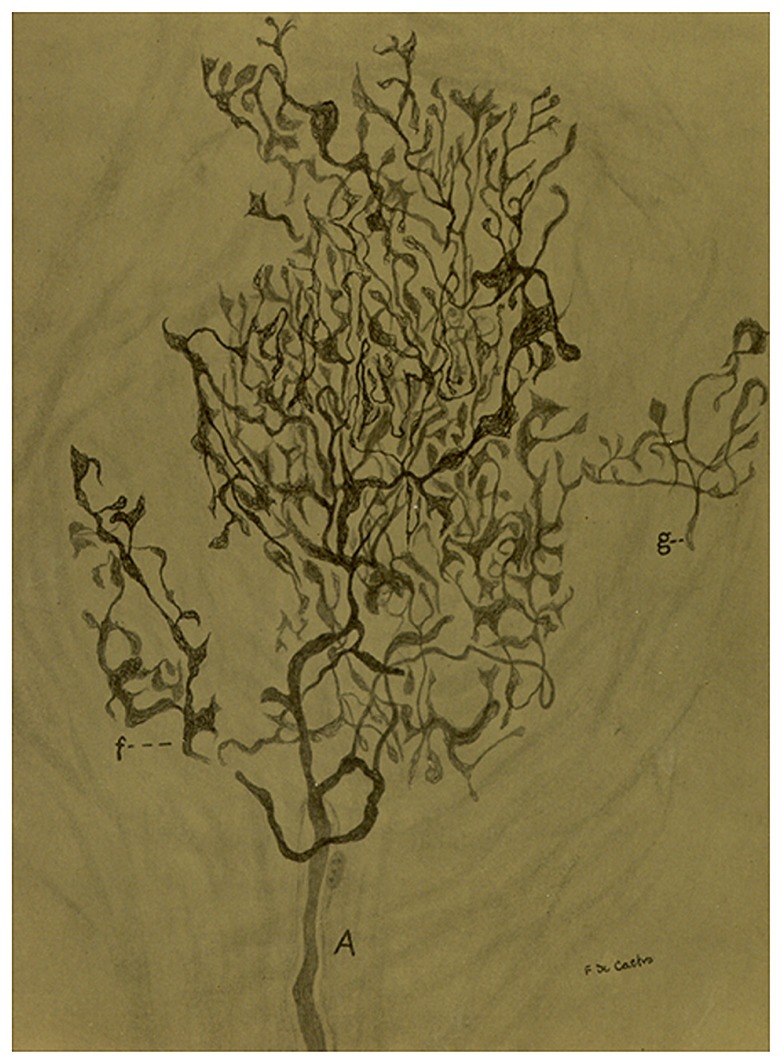
**Sensory ending in a tangential section in the carotid sinus of a man**. (A) Thick myelinated axon that arborises in a rather beautiful pattern with different details in the terminals. (f) A branch of the main fiber. (g) Nerve terminal in racket. The preparation was stained by the Cajal’s reduced silver nitrate method (in de Castro, 1928; courtesy of Fernando-Guillermo de Castro from the Archive Fernando de Castro).

The topography and density of the innervation present minimal variations, other than those derived form the anatomical localization of the elastic segment in cows. There is a band or belt around the elastic segment where the sernsory terminals concentrate, although the limits of the disposition of the endings are not always precise, with zones in the upper and lower ends in which the innervation is less dense. In the cow, the zones of maximal density of innervation are the internal zones of the occipital artery at the angle formed by the occipital artery and the external carotid artery. There is later in the paper an ample, meticulous, and beautiful description of the morphology of the sensory fibers and terminals in the carotid sinus of men and other mammals that we will present in a very condensed manner. The innervating fibers, usually myelinated of different diameters, have their origin in the sensory ganglia of the vagus or glossopharyngeal (note that in the 1926 paper he sustained that they were vagal fibers) and from the periglandular plexus reach the sinus by its superior pole. In the carotid sinus of men, de Castro distinguishes at least two types of branching pattern for the fibers on arriving to the adventitia: (1) diffuse arborisations which are originated from small or middle sized myelinated fibers and follow a rather regular division pattern giving branches of first, second, third and even larger orders; in their trajectories they might have small varicosities and in the terminals capricious forms (leaves, rackets, mallets,…). (2) circumscribed ramifications which are originated from medium to thick sized myelinated fibers and follow an arborisation pattern like grapevines or bushes; branches are spiny and zigzagging, and the terminals draw small triangular structures (**Figure 5**). In the rest of the mammals studied, the same two types of branching patterns are distinguishable having an overall complexity that parallels the size of animals. de Castro shows a clear parallelism between the large sizes of the architecture of the sensory fibers their arborisations and terminals in the occipital artery of the cow vs. the much simpler pattern of branching of the sinus of the mouse.

Finally, de Castro devotes a small section to describing the relationships between the disposition of the sensory branches and the organization of the adventitia of the carotid sinus area: Knowledge of this relationship, he states, would aid understanding the mechanism of activation of the sensory fibers when changes in blood pressure occur. As a general rule de Castro observes that the fascicles of collagen fibers in the adventitia have a parallel disposition following the longitudinal axis of the sinus region and the branches of the fibers are disposed among those fascicles. On occasion this regular pattern is lost due to the existence of oblique fascicles of conjunctive tissue, being in these areas where the zigzagging branches of the sensory fibers are housed. de Castro reasoned that natural stimulation of the sensory apparatuses would occur by their compression between the collagen fascicles when the wall of the arteries becomes distended by the arterial pressure. In this manner the sensory innervation of the carotid sinus area would be transmitting constantly the level of the arterial blood pressure to brain stem centers and, as the experiments of Hering have demonstrated, an increase in the pressure of the area would trigger the sinusreflex.

**FIGURE 6:**
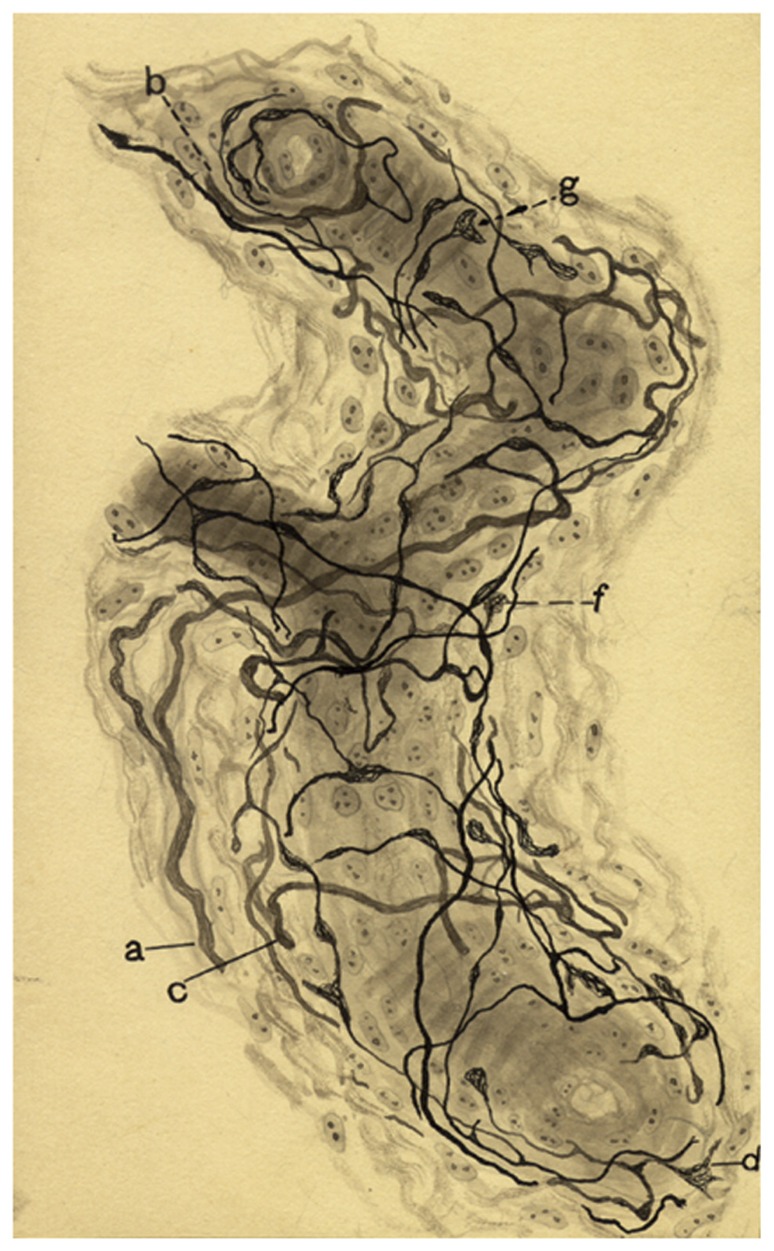
**Sensoy nervous plexus in an artery of small caliber from a dog of two months**. (a,b,c) Myelinated axons. (d) Varicose nerve terminal, (g,f) Terminal meniscus. The preparation was stained by the Cajal’s reduced silver nitrate method (in de Castro, 1928; courtesy of Fernando-Guillermo de Castro from the Archive Fernando de Castro).

The next section in the 1928 article is devoted to the sensory innervation of the intraglomic vessels. de Castro expanded the description of the sensory innervation of intraglomic vessels given in 1926. The fibers and their terminals would be distributed in small arteries and arterioles and even in precapillaries where the sinusoids of the CB are initiated. de Castro restates de simplicity of the innervation and the general branching pattern of the nerve fibers in comparison to those present in carotid sinus and provides some magnificent images obtained in longitudinal views of intraglomic vessels (**Figure 6**).

Once again de Castro calls attention to the sympathetic innervation of the intraglomic arterial vessels that would come mostly from intraglomic or periglomic sympathetic neurons receiving their preganglionic innervation via the ganglioglomerular nerves. From a functional point of view he declines his previous 1926 beliefs: that innervation of the intraglomic vessels would represent the afferent arm of a reflex which via the sympathetic innervation would promote profuse secretion of the purported carotid gland and that the sensory innervation of the CB vessels would not have a complementary role to that of the carotid sinus. In this 1928 study de Castro denervated the carotid sinus region by careful application of phenol solutions that would destroy the carotid sinus-internal carotid artery nerve endings, and this denervation preserved, albeit attenuated, the typical systemic depressor response when the pressure in the cannulated common carotid artery was augmented; he concluded that the sensory innervation of the intraglomic vessels is functionally complementary to the innervation of the carotid sinus.

In spite of these observations, the existence of a reflex loop initiated in the sensory innervation of intraglomic arteries and arterioles and closing at the sympathetic innervation of the same vessels cannot be dismissed. Obviously this reflex would not control the secretion of the carotid gland, because the CB is not a gland, but it could control the chemosensory activity in the CSN. This reflex loop would represent an efferent control of the CB function, in addition to the above described efferent pathway. Although we do not yet know the circumstances in which this sympathetic based efferent control would work there are some well established facts in that regard. For example, Eyzaguirre and Lewin (1961) demonstrated that stimulation of either the preganglionic sympathetic trunk or the ganglio-glomerular nerves produced an increase in the chemosensory activity (non-baroreceptor sensory activity recorded in the CSN) that was attributed to a decrease in CB blood flow (and therefore to a decrease in CB O_2_ supply) because sympathetic stimulation lacked effects in the isolated superfused preparation. The observation that stimulation of the sympathetic supply to the CB augments chemosensory activity has been ratified by many laboratories, but the doubt remains: under which circumstances are the sensory nerve endings of intraglomic vessels activated to initiate the reflex loop? The truth is that we do not know. Lahiri’s laboratory (Lahiri et al., 1986) showed that the responses of the ganglioglomerular nerves were associated with the respiratory responses to hypoxia and hypercapnia, so it could be speculated that the changes in blood flow that occur in the CB during its physiological stimulation contributes, via the sensory innervation of intraglomic vessels to set the sympathetic tone directed to the CB vessels and thereby to fine tuning the chemosensory activity in the organ.

The next section of the article is entitled New facts on the innervation of glomus caroticum (the CB) and its likely function. de Castro splendidly summarizes his previous findings along with many of the experimental approaches he employed to discover the function of the CB. He states that the CB is not the origin of the sinus reflex, the CB does not meet the criteria to be considered a paraganglion, in sum, as he had stated at the beginning of this 1928 paper: “up to now I have being unable of finding a function for the CB.” Aiming for a definitive answer, de Castro performed two physiological and anatomical experiments. In the first experiment performed in cats he approached the phossopharyngeal through the bulla tympanica and sectioned the nerve placing a thread of silk in the distal stump to handle it adequately; he later sectioned the peripheral branch of the glossopharyngeal going to the tongue thereby obtaining an easy-to-handle preparation with the intact CSN and a small, much thicker segment of the glossopharyngeal. When he stimulated this CSN preparation while recording the arterial blood pressure in the femoral artery no changes were observed, but if he stimulated the intact CSN without the prior proximal sectioning of the glossopharyngeal he obtained the Hering’ s reflex in all its clarity. He concluded that the stimulation of the functional nerve of the CB had not provided any insight as to the physiological significance of organ. The second experiment performed in 17 cats and three dogs was more decisive. The experiments consisted in the intracranial sectioning of the roots of the glossopharyngeal (9th) and vagus (10th) and occasionally also of the accessory (11th) nerves. 5–12 days after the surgery the glossopharyngeal and the vagus in their extracranial segments as well as the area of the division of the common carotid artery were collected for histological analysis. It was found that in glossopharyngeal there were a very small number of degenerated fibers with an even smaller number in the CSN, so that the appearance of the CSN was essentially normal. The CB cells exhibit an intact innervation after the intracranial section of the glossopharyngeal, there being no differences, other than the expected vascular congestion, between the operated and the contralateral control CB. These findings obliged de Castro to conclude that the trophic centers (the neuronal somas) of the axons innervating the CB cells are located in the sensory ganglia of the glossopharyngeal, i.e., the petrosal ganglion, and not in the brain stem as he had concluded in his previous study. To assure that the surgical procedures and therefore the conclusions were correct, de Castro stimulated electrically the glossopharyngeal and the CSN of the operated side and no response was obtained, while on stimulating the contralateral side he obtained the typical depressor response.

It is in the conclusion of this section where de Castro demonstrates unequivocally the sensory nature of the CB and where he creates the concept of chemoreception. de Castro wrote: “In sum, the glomus caroticum is innervated by centripetal fibers, whose trophic centers are located in the sensory ganglia of the glossopharyngeal nerve, and not by centrifugal secretory fibers as is the case in the glands. The CB is a special sensory organ, whose function was not an enigma. We hypothesized that the CB is a sensory organ devoted to perceive some qualitative modifications of the blood, and not a system devoted to detect the variations of the blood pressure; this last function residing in the carotid sinus and also in part in the arteries and arterioles of the CB itself.”

In the following section of the paper that he entitles General considerations and discussion, de Castro devotes great efforts to refute the notion of the CB as the origin of the Hering’s reflex, because it was a notion that was gaining adepts in spite of the really conclusive experiments of Hering and de Castro himself. He also refuted the notion of the CB as a (sympathetic) paraganglion because of the dubious (negative) nature of the chromaffin reaction and to the fact that some authors found that the CB extracts caused hypotension while those of the adrenal gland caused an elevation of blood pressure. He also refuted his previous notions of the CB as a special gland non-classifiable among the paraganglion group. Finally, he refused to accept an involutional or rudimentary functional significance to the CB. However, he maintained the notion of a possible cooperative significance of the innervation of the CB vessels in the final setting of the Hering reflex.

Later de Castro reiterates that the intracranial section of he glossopharyngeal has made evident that the innervation of the CB cells is sensory and therefore the CB as a whole represents a sensory organ, the only one presently known, responsible for detecting changes in the qualitative composition of the blood, and possibly, using a reflex pathway might affect the functional activity of other organs. Certainly de Castro did not know the nature of stimulus triggering the reflex nor the targets of the reflex itself, but he clearly outlined the CB chemoreflex. de Castro conceives the parenchymatous elements of the CB (i.e., the chemoreceptor cells) as having two poles, one vascular and other nervous. Through the vascular pole the cells are in a close relationship with the sinusoidal capillaries that surround the cell clusters to taste the blood composition. By the nervous pole the cells are innervated by polymorphic nerve endings. The nerve endings or nerve fibers would not be stimulated directly by the blood, but rather by the mediation of the parenchymatous cells whose products of secretion would activate the stimulation of the centripetal fibers (see also de Castro, 1951).

Certainly, de Castro in these two articles (1926 and 1928) here summarized has built a frame, a circuit through which neural orders must circulate to contribute to maintain the internal equilibrium of the organisms, the adjusting to physiological processes and a potential mechanism of defense in disease. It remained to be established how the circuit is put into motion, how it is activated, how it works, where it impinges to contribute to homeostatic equilibrium. These aspects of the CB physiology started to be developed almost immediately in the laboratory of Corneille Heymans, being the merit of the Belgian authors the discovery of the qualitative changes in the composition of the blood that the chemoreceptor cells detected (see the title of the paper) as well as the description of lungs as the main target of the chemoreflex (Heymans et al., 1930). To fully appreciate the significance of de Castro’s work we copy as a foot note a fragment of the Award Ceremony Speech given by Prof. G. Liljestrand in 1938 when Corneille Heymans received the Nobel Prize in Physiology and Medicine^2^.

## PRESENT STATUS AND PERSPECTIVES IN CB MECHANISMS AND POTENTIAL PATHOPHYSIOLOGICAL SIGNIFICANCE

The systemic targets of the carotid chemoreflex had largely been described by the middle of 1980s (Fitzgerald and Lahiri, 1986). Physiologically, the CB plays a homeostatic and adaptive role. Chemoreceptor cells are naturally stimulated by hypoxia and hypercapnia and the secretion of chemoreceptor cells, the release of their neurotransmitters, activate the sensory nerve endings of the CSN which via the glossopharyngeal nerve carry the activity to the brain stem; the information incoming from the CB is integrated at this level to reflexly generate proportional ventilatory and cardiocirculatory responses aiming to normalize blood gases and to minimize the deleterious effects of their alterations. Aside from the respiratory and cardiocirculatory systems the reflex initiated at the CB has many more targets, being capable of initiating, whether directly or indirectly, a large array of systemic responses that help to cope with the original unbalances that stimulated the CB (Fitzgerald and Shirahata, 1997). However, in recent years a new concept is emerging on assigning to the CB a great significance in pathological processes. It is thought that processes such as the origin and development of hypertension particularly when associated to the obstructive sleep apnea syndrome, insulin resistance and type II diabetes, heart failure and obesity share increased sympathetic activity as a pathophysiological mechanism; this sympathetic hypertony being originated in part by afferent signals emerging from the CB. In turn, these new concepts are creating the notion that the inhibition of the CB activity, the CB denervation or even the surgical removal of he CB might have a favorable impact on the morbidity and mortality of those processes (Paton et al., 2013; Ribeiro et al., 2013).

**FIGURE 7:**
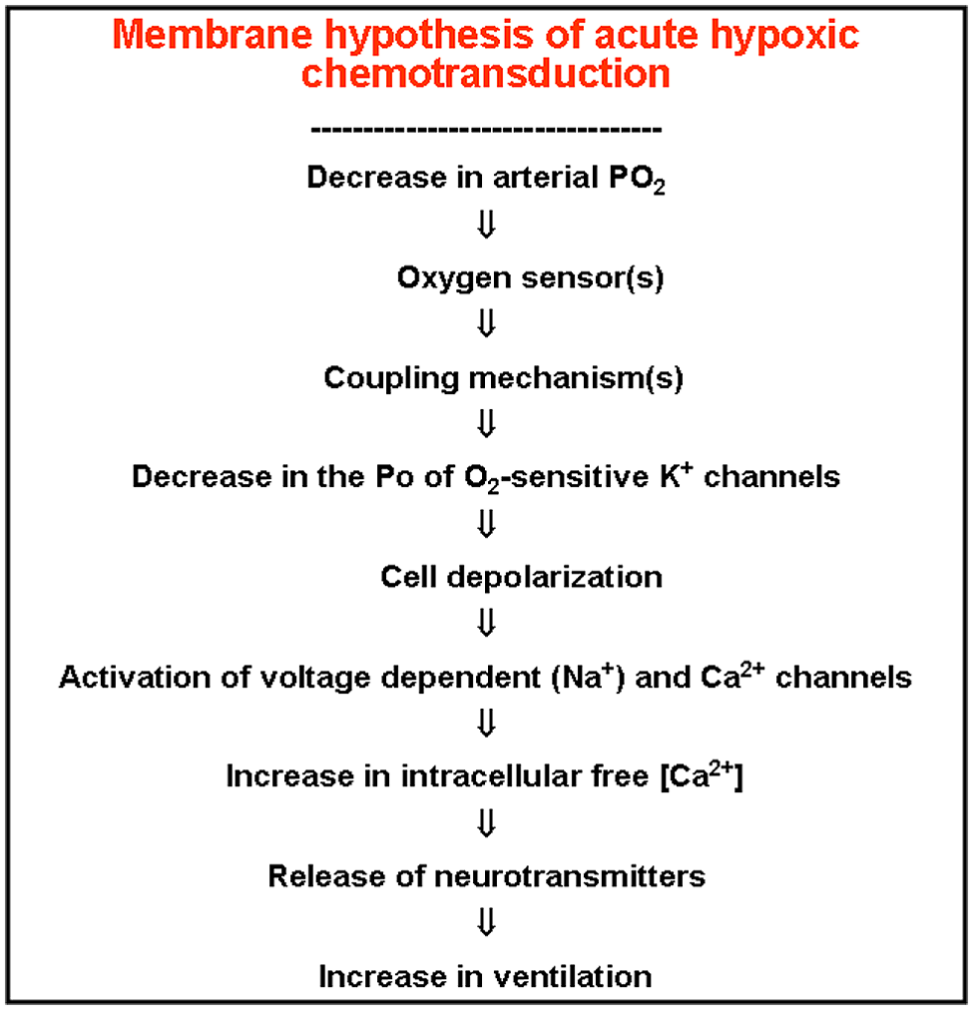
**The membrane model of acute hypoxic transduction proposes the cascade of events depicted in the figure**. On lowering PO_2_, an O_2_^-^ sensor, hypothetically located in the plasma membrane, would experiment a conformational (and reversible) change, which transmitted to oxygen-regulated K^+^ channels would cause modifications in their kinetic properties, resulting in a decrease in their opening probability. The ensuing depolarization activates voltage dependent Ca^2^^+^ channels and entry of Ca^2^^+^ in the cells triggering the exocytotic release of neurotransmitters, increase in CSN action potentials and in ventilation (redrawn from González et al., 1992).

One of the most fundamental questions in CB physiology was, and to some extent continues to be, the mechanisms involved in the O_2_ sensing in chemoreceptor cells. Literature up to the middle 1980s was full of imaginative hypotheses with few supportive experimental data (Fidone and Gonzalez, 1986). Our laboratory has been collecting experimental observations indicating that hypoxia must depolarize chemoreceptor cells, because it promoted the release of dopamine from the cells by a process that was Ca^2^^+^-dependent and sensitive to antagonists of voltage operated Ca^2^^+^ channels. In a search for mechanisms capable of producing the depolarization required to activate the voltage-operated Ca^2^^+^ channels we found that rabbit carotid chemoreceptor cells expressed K^+^ channels that were selectively inhibited by acute hypoxia (Lopez-Barneo et al., 1988). This finding originated the formulation of the so-called membrane hypothesis for hypoxic chemotransduction that we have formulated as shown in the **Figure 7**. Although the expression of O_2_-sensitive K^+^ channels was confirmed in all studied species by different laboratories, and therefore the membrane hypothesis gained wide support, there are many fundamental facts that remain unanswered. For example, how hypoxia couples to K^+^ channels to alter their kinetic properties? Since there is a marked inter-species diversity O_2_-sensitive channels it would appear that chemoreceptor cells expresses a unique O_2_ sensor capable of interacting with the different channels. If the existence of a sensor is accepted, how does it interact with the channels? Are the beta-like regulatory subunits required? (see Pérez-García et al., 1999).

As Conde and Peers (2013) discuss, the discovery of those very basic mechanisms go beyond the pure academic interest of expanding knowledge. Thus, if as we have described in preceding paragraphs the hyperactivity of the CB is involved in the pathogenesis of several clinically relevant processes, the adequate manipulation of the basic machinery involved in chemoreceptor cell activation might represent the most rational approach for their treatment. The membrane model for chemoreception, as depicted in **Figure 7**, was formulated over twenty years ago (González et al., 1992), and consequently there have been significant additions to it, being worth noting the proposal of several mechanisms acting as primary oxygen-sensors and/or elements coupling the sensor to K^+^ channels (Gonzalez et al., 2010). Second messengers capable of modulating the flow of information in the transduction cascade have also being described with the cAMP-EPAC system having a particular relevance as a positive regulator of the hypoxia triggered responses (Rocher et al., 2009). Finally, among others, Prabhakar’s laboratory has generated a significant number of studies showing that hydrogen sulfide, which is produced in chemoreceptor cells, is capable of stimulating the CB by a Ca^2^^+^ dependent mechanism, being proposed that maxi-K^+^ channels are the potential targets-effectors of this gasotransmitter (Prabhakar and Peers, 2014).

Finally, another aspect of the general physiology of the CB that has received great attention but it is not satisfactorily resolved, is, using de Castros’s words, the product of secretion of chemoreceptor cells that stimulates the chemosensory nerve endings, i.e., the nature of the neurotransmitter(s) responsible for setting the activity in the CSN. Historically, acetylcholine was the initially proposed and most firm candidate to represent the key “conveyor” of information from chemoreceptor cells to the chemosensory terminals which at some early point in the nerve fiber would generate conducted action potentials driving the information to the brainstem. The cholinergic hypothesis was based on experimental observations obtained in the cat, and was rejected because while acetylcholine and nicotinic agonists were powerful chemostimulants and classical nicotinic antagonists blocked these responses, they were ineffective to eliminate the chemosensory activity elicited by natural stimuli. The progressive discovery of neurotransmitter systems in the central nervous system, including the neuropeptides, and the appearance of the concepts of co-storage and co-release of neurotransmitter substances invaded the CB field as chemoreceptor cells appeared to express every single neurotransmitter system. In the sensory synapse established between chemoreceptor cells and the CSN terminals, as is the case in every synapse studied, the existence of postsynaptic, presynaptic and extrasynaptic receptors for the putative neurotransmittes was recognized. This complex neurochemical arrangement, added to the intricate anatomical organization of the CB, has made it very difficult to interpret the classical pharmacological experiments and to demonstrate the criterion of identity of action of any candidate neurotransmitter. Difficulties have been amplified by the existence of genuine differences among species, both in the concentrations of one or another neurotransmitter (e.g., dopamine vs. norepinephrine) and dominance of one or another receptor subtype (nicotinic vs. muscarinic). Many laboratories have their particular bias in the study of one or another neurotransmitter system, and therefore articles giving a wide coverage to neurotransmission in the CB are not abundant. Among those articles, our 1994 comprehensive review (Gonzalez et al., 1994) has a broad section which compiles what could be considered classical findings on every neurotransmitter system. In our review we discuss at length the great controversy existing in the understanding of the function of dopamine in the CB. Yet there are three facts that we consider firmly established: (1) Although in most species dopamine is considered an inhibitory neurotransmitter-neuromodulator, in the rabbit, species in which we have carried out most of our experiments, dopamine appears to fulfill the criteria to be considered an excitatory neurotransmitter at the synapse between chemoreceptor cells and chemosensory nerve terminals (see the article by Iturriaga et al., 2009); (2) in every species studied there is a reasonable parallelism between the intensity of natural stimulation and the amount of dopamine released by chemoreceptor cells, making the measurement of the release of dopamine a very valuable index of the activity of chemoreceptor cells, both in intact organs or in isolated cells, and (3) the dopaminergic (catecholaminergic) trait, i.e., the immunohistochemical positiveness to tyrosine hydroxylase, is probably the most universally accepted criterion to identify chemoreceptor cells, both in organ sections or in tissue culture. In more recent times the interest for the study of neurotransmitters in the CB has not weakened. We want to mention the recent study by Conde et al. (2012) showing that in the rat ATP and adenosine are key neurotransmitters involved in hypoxic CB chemotransduction, with a more relevant contribution of adenosine during mild hypoxia, and a more prominent role for ATP in high-intensity hypoxia. However, there is no doubt that Colin Nurse’s laboratory has been leading the study of neurotransmission in recent years (Nurse, 2010). This laboratory using rat as their experimental species developed the aforementioned simplified co-culture preparation in which chemoreceptor cells or cell clusters were plated together with neurons dissociated from the petrosal ganglion which in a lapse of time of less than one week formed functional synapses. Under these conditions it was possible to record in the petrosal neurons spontaneous postsynaptic-like potentials and burst of action potential when the preparation was stimulated by a hypoxic pulse. In this preparation it was possible to study both in the postsynaptic element (the petrosal neuron) and in the presynaptic element (the chemoreceptor cells) the effects of agonists and antagonists of putative neurotransmitters. The conclusions attained with this preparation are that ATP and acetylcholine released by chemoreceptor cells and via P2X_2/3_ and nicotinic receptors would act as excitatory neurotransmitters in the postsynaptic element of the chemoreceptor cell-sensory terminal synapse. GABA would also be released by chemoreceptor cells and via GABA_A_ receptors also present in the sensory nerve endings would be an inhibitory neurotransmitter. Serotonin and adenosine would act on autoreceptors in chemoreceptor cells as positive modulators while GABA, dopamine and histamine would be negative modulators of chemoreceptor cell activity. The undeniable merits of Nurse’s experiments might have a potential handicap, namely that in the co-culture conditions some neurotransmitters and their receptors might change their expression. For example, while the findings of Nurse’s laboratory unequivocally would indicate that acetylcholine is being release form chemoreceptor cells, Gauda’s laboratory (Gauda et al., 2004) working with rat pups from 2 to 28 days of postnatal age showed that tyrosine hydroxylase positive chemoreceptor cells of freshly obtained CB tissue do not express the most reliable cholinergic markers, choline acetyltransferase and vesicular ACh transporter studied by semiquantitative in situ hybridization histochemistry and immunohistochemistry.

In conclusion, the working program outlined by de Castro on his discovery of arterial chemoreceptors still represents the path to follow: we must deepen our understanding of the molecular mechanisms used by chemoreceptor cells to “taste” hypoxia and hypercapnia, we must devise new preparations, probably based in imaging techniques, that allow faithful study of the communication between chemoreceptor cells and the sensory nerve endings, and finally, we must attempt to understand how the blood flow of the CB is regulated and how it affects the functioning of the arterial chemoreceptor. The fulfillment of this program would certainly allow the understanding of the CB malfunctions and the correction of CB-linked pathological processes.

## Conflict of Interest Statement

The authors declare that the research was conducted in the absence of any commercial or financial relationships that could be construed as a potential conflict of interest.

## Footnotes

1 The historical account we are providing here has been made after a careful reading of Adams book. Adams W. E. The comparative morphology of the CB and carotid simus. Charles C Thomas, Publisher: Springfield, Illinois, USA, 1958.

2 http://www.nobelprize.org/nobel_prizes/medicine/laureates/1938/press.html… The glomus … has been considered … a sort of endocrine gland similar to the medulla of the suprarenal glands. de Castro (1928) demonstrated that the anatomy of the glomus could no be compared to that of the suprarenal medulla. de Castro suggested that the glomus was an organ whose function was to react to variations in the composition of the blood … an internal gustatory organ with special “chemo-receptors.” Heymans et al. (1930) undertook to find out whether these supposed chemo-receptors were responsible for the respiratory reflexes produced by modifications in the composition of the blood.
